# Development of an Immunochromatographic Test Strip for Detection of Cholera Toxin

**DOI:** 10.1155/2013/679038

**Published:** 2013-11-07

**Authors:** Eiki Yamasaki, Ryuta Sakamoto, Takashi Matsumoto, Fumiki Morimatsu, Takayuki Kurazono, Toyoko Hiroi, G. Balakrish Nair, Hisao Kurazono

**Affiliations:** ^1^Division of Food Hygiene, Department of Animal and Food Hygiene, Obihiro University of Agriculture and Veterinary Medicine, Nishi 2-11 Inada-cho, Obihiro, Hokkaido 080-8555, Japan; ^2^R&D Center, Nippon Meat Packers, Inc., 3-3 Midorigahara Tsukuba, Ibaraki 300-2646, Japan; ^3^Division of Clinical Microbiology, Saitama Institute of Public Health, Saitama 338-0824, Japan; ^4^Translational Health Science and Technology Institute, Plot no. 496, Phase III, Udyog Vihar, Gurgaon, Haryana 122016, India

## Abstract

Because cholera toxin (CT) is responsible for most of the symptoms induced by *Vibrio cholerae* infection, detection of CT is critical for diagnosis of the disease. In this study, we constructed an immunochromatographic test strip for detection of CT (CT-IC) with polyclonal antibodies developed against purified recombinant whole CT protein. The detection limit of the CT-IC was 10 ng/mL of purified recombinant CT, and it could detect the CT in culture supernatant of all 15 toxigenic *V. cholerae* isolates examined, whereas no false-positive signal was detected in all 5 nontoxigenic *V. cholerae* isolates examined. The specificity of the CT-IC was examined with recombinant heat-labile toxin (LT), which shares high homology with CT, and it was revealed that the minimum detection limit for LT was 100 times higher than that for CT. In addition, *lt* gene-positive enterotoxigenic *Escherichia coli* (ETEC) was examined by CT-IC. The false-positive signals were observed in 3 out of 12 ETEC isolates, but these signals were considerably faint. The CT-IC did not develop false-positive signals with all 7 *V. parahaemolyticus* isolates. These results showed the high specificity of CT-IC and the feasible use of it for the detection and surveillance of toxigenic *V. cholerae*.

## 1. Introduction

Cholera remains a major public health problem especially in developing countries. The seventh pandemic of cholera which began in 1961 is still ongoing. In recent years, cholera cases have steadily increased. In 2011, the cholera cases reported to WHO were from 58 countries and accounted for 589,854 cases including 7,816 deaths [1]. The most recent epidemic is striking in Sierra Leone where over 20,000 cases including 280 deaths had been reported before October 2012. Furthermore, the actual number of cholera cases is assumed to be much higher than those reported. This discrepancy is attributed to the lack of dissemination of effective surveillance system. Because underreporting or underestimation impedes implementation of sufficient control measures, further improvement in surveillance system, which contributes largely to determine the true number of incidences, is still required.

Currently, several diagnostic procedures including the “gold standard” of culture test and rapid diagnostic tests are available for *V. cholerae* detection [2]. In culture test, alkaline peptone water (APW) and TCBS are commonly used as enrichment and selective media, respectively. As many have noted, the cultivation test is time-consuming, but it has the advantage of being able to isolate the causative bacterium which can be used for further characterization. On the other hand, utility of various rapid diagnostic tests such as polymerase chain reaction (PCR), quantitative PCR (qPCR), loop-mediated isothermal amplification (LAMP), enzyme-linked immunosorbent assay (ELISA), reverse passive latex agglutination test (RPLA), and immunochromatographic test (IC) has been demonstrated. These rapid methods facilitate timely, in some cases, on-site responses. And, the rapid detections in early stage of epidemic allow quick triggering of control measures. In the case of diagnosis of cholera, after or along with the detection of bacterium, verification of cholera toxin (CT) production is required because only the *V. cholerae* which can produce CT is responsible for cholera symptoms such as acute “rice water” diarrhea. Some detection methods for toxigenic* V. cholerae* have been described previously. The approaches to assay for CT can be divided in terms of features to be detected: (1) bioassay including rabbit ileal loop test, rabbit skin test, and cultured CHO cell assay, (2) immunoassay including ELISA and RPLA, and (3) DNA-based assay including PCR, qPCR, DNA hybridization, and LAMP [3, 4]. Combined use of more than one detection method would be required to increase the accuracy of a diagnosis. At that time, combination of different target analytes; for example, immunoassay which detects the existence of toxin and DNA-based assay which detects the existence of toxin-coding DNA must be chosen.

While DNA-based assays may be more sensitive than immunoassays, the latter has an important advantage in the detection of extracellular bacterial toxin. Recently, some new methodology of immunoassay with extremely high sensitivity has been reported [5, 6]. However, IC is still one of the most commonly utilized immunoassays because it is rapid and very easy to conduct. In the present study, we constructed IC for CT detection (CT-IC). To increase sensitivity, we used the polyclonal antibodies established against whole toxin which contains both A (active) and B (binding) subunits. We demonstrated that the constructed CT-IC could detect CT in *V. cholerae* culture in which more than 10 ng/mL of CT was expressed.

## 2. Materials and Methods

### 2.1. Bacterial Strains

Fifteen *ct* gene-positive *V. cholerae* isolates (7 O1 El Tor Ogawa, 6 O1 El Tor Inaba, and 1 each of O139 and O141) and 5 *ct* gene-negative *V. cholerae* strains (2 each of O1 El Tor Ogawa and O1 El Tor Inaba and 1 of O139) were kindly provided by Saitama Institute of Public Health, Saitama, Japan. These strains were isolated in Japan from 1993 to 2007. Each strain was individually isolated from diarrhea patients including 15 traveler's diarrhea patients who traveled to India, Philippines, China, Thailand, Egypt, Greece, or Iran, and 3 patients who are infected domestically, and 2 patients lacking detailed information (Table 1). Twelve enterotoxigenic *E. coli* isolates were laboratory stock strains which were isolated from stools of diarrhea patients in India. Seven *V. parahaemolyticus* strains were the strains isolated from food samples or patients in Hokkaido, Japan.

### 2.2. Preparation of Purified CT and LT

The laboratory stocks of anti-CT antiserum and anti-LT antiserum were used for preparation of anti-CT IgG conjugated column and anti-LT IgG conjugated column. These antisera stocks were prepared according to a protocol previously described [7] by using purified recombinant CT and LT which were purified as described in a previous report [8] as an antigen. For antiserum conjugated column preparation, the laboratory stocks of antisera were coupled to HiTrap NHS-activated HP (5 mL, Amersham Biosciences) according to the manufacture's instructions.

For CT and LT purification, previously constructed *E. coli* MC1016 (pKTJ5-15x) strain and *E. coli* HB101 (pKTN1003b) strain in which recombinant CT and LT were overexpressed respectively [8], were inoculated into LB broth supplemented with ampicillin (50 *μ*g/mL). After overnight cultivation at 37°C, the bacterial cells were collected by centrifugation, resuspended in PBS, and then disrupted by sonication. The obtained cell sonicates were centrifuged to obtain soluble fractions. Proteins in the soluble fractions were precipitated with 80% AmSO_4_, resuspended in PBS, treated with RNase and DNase, and then applied to anti-CT antisera conjugated column and anti-LT antisera conjugated column. After the columns were washed with PBS, recombinant CT and LT proteins were eluted with 0.1 M glycine-HCl, pH3.0. After dialysis, purified samples were condensed by using of Centricon YM-10 (Merck Millipore). Purities of the samples were analyzed by SDS-PAGE and CBB staining. Concentrations of the purified samples were determined by Bio-Rad Protein Assay Dye Reagent Concentrate (Bio-Rad) by using bovine serum albumin as a standard.

### 2.3. Preparation of IgG Specific for CT

Anti-CT polyclonal antibodies were raised in a rabbit against purified recombinant CT according to a protocol previously described [7]. In brief, purified CT (100 *μ*g protein in 1 mL of PBS) was mixed with the same volume of complete Freund's adjuvant and injected intradermally into 7-week-old Slc:JW/CSK rabbit. Three weeks after the first injection, the rabbit was boosted with the same amount of antigen mixed with complete Freund's adjuvant. One week after the second injection, 50 *μ*g of the antigen without adjuvant was injected, and from then on, the same amount of the antigen without adjuvant was injected once a week. Antibody titers were determined by an Ouchterlony double immunodiffusion test with purified recombinant CT. Rabbits were bled 9 weeks after the first injection. Whole blood collected from immunized rabbit was left at room temperature for 4 hours, incubated at 37°C for 2 hours, and kept standing under refrigeration overnight. Clear serum obtained after centrifugation of the blood clot at 3,000 rpm for 10 min was used for the purification of anti-CT specific IgG. After the proteins were precipitated by adding of Na_2_SO_4_ (1.27 M) to the serum, the precipitate was resuspended in phosphate buffer (0.02 M NaH_2_PO_4_, 0.02 M Na_2_HPO_4_, pH 7.0) and dialyzed thoroughly against the same buffer. The preparation was loaded onto 1 mL HiTrap NHS-activated HP coupled with purified recombinant CT. After incubation of the column at 4°C for 2 hours, the column was washed with 10 bed volumes of phosphate buffer. And then, the bound CT-specific IgG was eluted with 0.1 M glycine-HCl, pH 3.0. Elute was monitored by measuring the absorbance at 280 nm. After elute was neutralized by adding of Tris base (0.1 M), the fractions containing CT-specific IgG were dialyzed thoroughly against PBS. Protein concentration for purified IgG specific for CT was determined by using of previously measured molar-absorbance coefficient.

### 2.4. Immunochromatographic Test

The test strip was prepared according to a protocol previously described [9]. For immunochromatographic test, 100 *μ*L of the samples was loaded onto the test strip placed on horizontal table, and after migration of the sample through the membrane for 15 min at room temperature, the appearance of red lines at the test (T) zone and the control (C) zone was analyzed. The results with the appearance of red lines at both the T and C zones were interpreted as positive detection of CT.

### 2.5. Quantitative Analysis of CT Production in Bacterial Culture Supernatant by Bead-ELISA

Preparation of quantitative bead enzyme-linked immunosorbent assay (Bead-ELISA) for CT and quantitative analysis of concentration of CT in culture supernatant were done as described previously [10]. To construct the Bead-ELISA, anti-CT-specific IgG prepared in this study was used. The concentrations of CT in culture supernatant were calculated with four-parameter logistic curve fit for points on the standard curve for purified recombinant CT. 

The bacterial culture supernatants were obtained after the organisms were cultured under AKI-SW condition [11]. Briefly, the organisms were cultured initially in stationary test tubes (height, 150 mm; diameter, 15 mm) for 4 h at 37°C, and then all the culture was transferred into 100 mL Erlenmeyer flasks. Subsequent cultivation was done at 37°C for 20 h with shaking (130 rpm). The amount of medium was maintained constantly at 10 mL. AKI medium (1.5% Bacto Peptone (Becton, Dickinson and Company), 0.4% yeast extract (Becton, Dickinson and Company) and 0.5% NaCI, and 0.3% NaHCO_3_) was used for all bacterial strains. The bacterial culture supernatant was obtained after centrifugation at 900 ×g for 5 min. 

## 3. Results

### 3.1. Detection Limit and Specificity of the Immunochromatographic Test Strip

**Figure 1:**
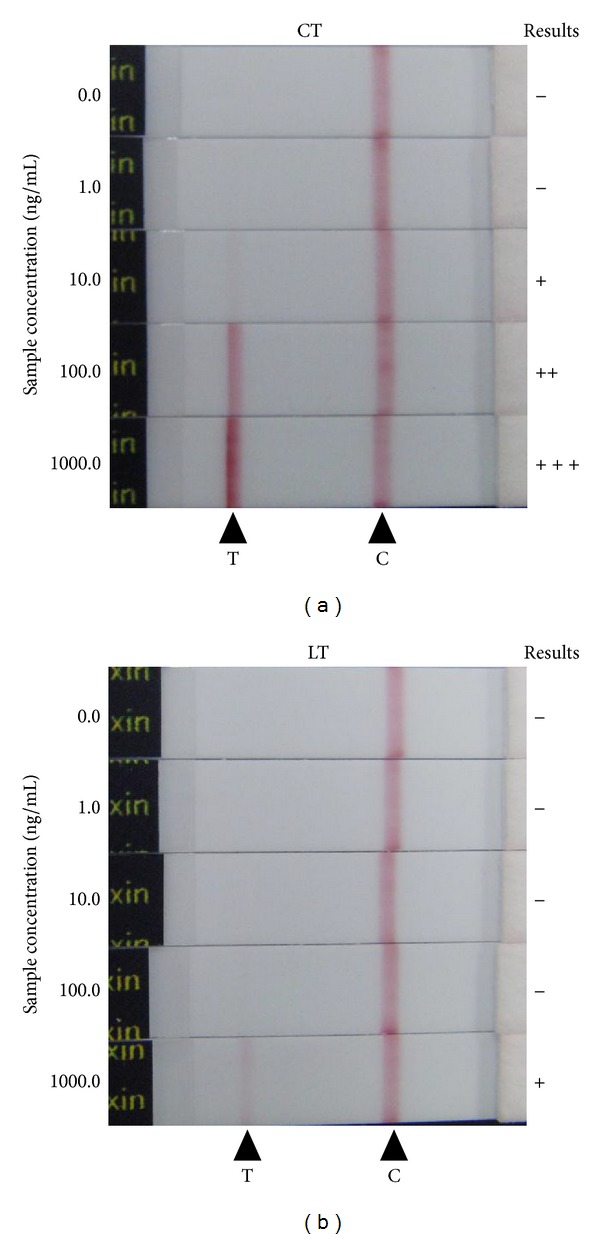
Reactivity of CT-IC with purified recombinant CT and LT. 0.1 mL of serial diluted purified recombinant CT (a) or LT (b) was applied to the test strips. After 15 minutes, development of red color at position for test (T) or control (C) lines was monitored. Concentrations of samples applied are indicated on right side of each strip. The “+++”, “++”, “+”, or “−” symbols are placed on the left side of the strips developing “strong,” “medium,” “faint,” or “no” bands at test lines, respectively.

To develop the high sensitive immunochromatographic test (IC), polyclonal antibodies were developed by using purified recombinant whole CT protein as an antigen. And, to increase the specificity, CT-specific IgG was isolated from antiserum by using purified recombinant whole CT conjugated column, and the obtained CT-specific IgG was used for construction of IC. We examined the lower detection limit of established CT-IC with 10-fold serial dilutions of purified CT (Figure 1(a)), and it was reveled that the CT-IC can detect as low as 10 ng/mL of purified CT within 15 min. 

Heat-labile toxin (LT) produced in enterotoxigenic *E. coli* (ETEC) was used for specificity verification because it shares around 80% amino acid homology with CT; therefore, it is known that LT is antigenically similar to CT. Examination with serial dilution of purified LT revealed that detection limit for LT was about 100 times higher than that for CT (Figure 1(b)), indicating that established test strip has high specificity.

### 3.2. Detection of CT from *V. cholerae *Culture

**Figure 2:**
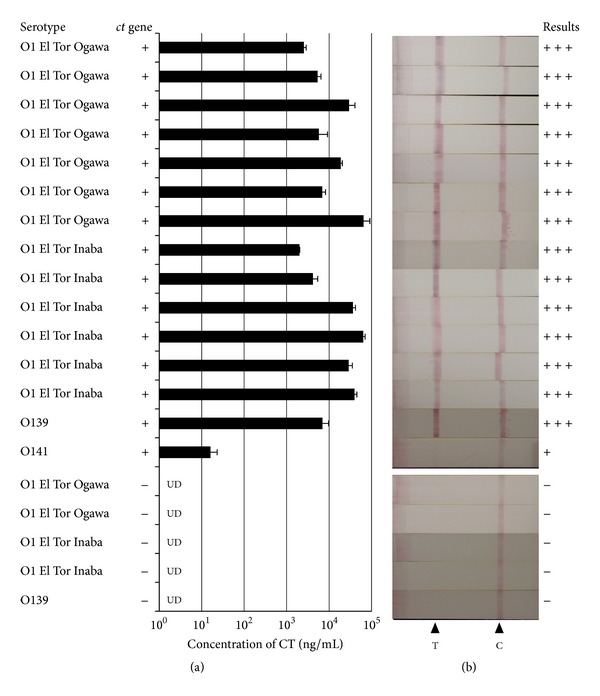
Ability of CT-IC to detect the toxigenic *V. cholerae *strains. Fifteen *ct* gene-positive *V. cholerae* isolates and 5 *ct* gene-negative *V. cholerae* isolates were cultured under AKI-SW condition, and then obtained supernatants of each culture were examined by quantitative Bead-ELISA specific for CT (a) or CT-IC (b). For quantitative analysis, data are means ± SD of values from three independent experiments. UD: undetectable. For IC, the “+++”, “+”, or “−” symbols were placed on the left side of the strips developing “strong,” “faint,” or “no” bands at test lines, respectively. T: test line, C: control line.

The optimal condition for CT production had been persistently investigated because the amount of CT produced in *V. cholerae* El Tor vary according to the medium used and culture conditions (i.e., temperature and aeration status) [11–14]. In this study, we used AKI medium with biphasic culture condition, that is, 4 h cultivation in a stationary test tube followed by 16 h cultivation in a shaking flask at 37°C (AKI-SW condition), because, under this condition, it was reported that the most of the *V. cholerae* El Tor strains could produce more than 10 ng/mL of CT proteins [13]. The quantitative analysis revealed that, in the case of *V. cholerae* strains we used in this study, 14 out of 15 *ct* gene-positive strains produced substantially high amount of CT (Figure 2(a)). And, even in the strain with low level of CT expression, concentration of CT in cultured supernatant was higher than the detection limit (10 ng/mL) of CT-IC. CT in culture supernatant of all 15 of *ct* gene-positive stains could be detected by CT-IC, whereas no false-positive signal observed in all 5 *ct* gene-negative *V. cholerae* strains (Figure 2(b)).

**Figure 3:**
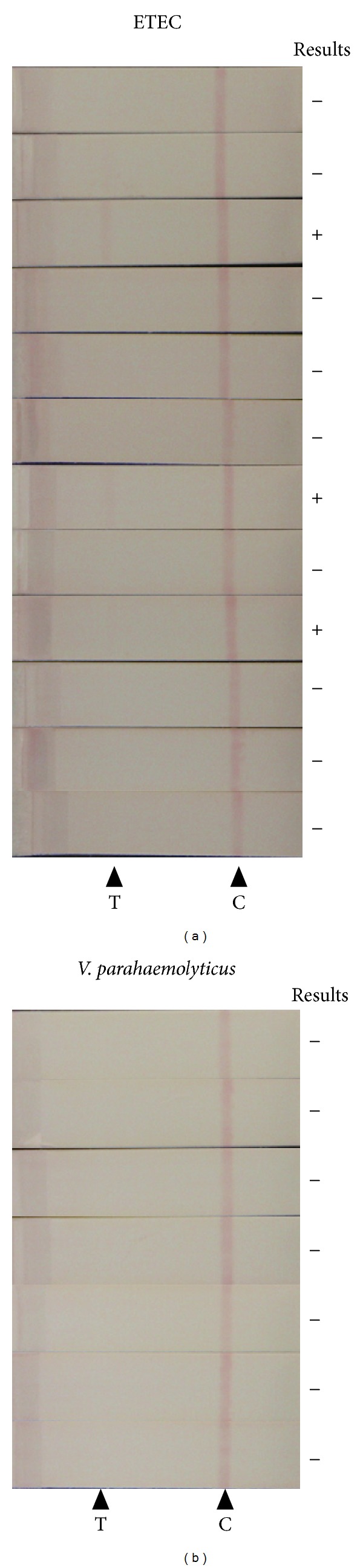
Reactivity of CT-IC with non-*V. cholerae *strains. Twelve ETEC isolates (a) and 7 *V. parahaemolyticus* isolates (b) were cultured under AKI-SW condition, and then obtained supernatants of each culture were examined by CT-IC. The “+” or “−” symbols were placed on the left side of the strips developing “faint” or “no” bands at test lines, respectively. T: test line, C: control line.

As indicated above, CT-IC can discriminate CT from LT and did not react with nontoxigenic *V. cholerae* strains. The specificity of CT-IC was further evaluated by examining the culture of bacteria other than *V. cholerae*. In this study, we examined the reactivity of CT-IC against cultures of ETEC and *V. parahaemolyticus*. As mentioned above, ETEC strains have the ability to express LT protein which shares high homology with CT. *V. parahaemolyticus* is the most frequently isolated species among genus *Vibrio* and is one of the most important food-borne pathogen worldwide. In the “gold standard” selective cultivation test, the same enrichment and selective media are usually used for isolations of *V. cholerae *and *V. parahaemolyticus*. Twelve *lt* gene-positive ETEC isolates and 7 *V. parahaemolyticus* isolates were cultured under AKI-SW condition, and the resultant culture supernatants were examined by CT-IC. As shown in Figure 3, although no strong signals could be detected, 3 out of 12 ETEC isolates gave weak false-positive signals. On the other hand, no false-positive signals were observed in all *V. parahaemolyticus* strains examined.

## 4. Discussion

Analysis of CT production is critical for accurate diagnosis of cholera because, even if *V. cholerae* is isolated from patient, we cannot attribute the symptoms to the isolated *V. cholerae* bacterium without verification of ability to produce CT. In this study, we constructed CT-IC with the lower detection limit of 10 ng/mL CT which is comparable to the detection limit of commercially available RPLA (1–3 ng/mL) [10]. Although both IC and RPLA are the simple and rapid detection techniques, the detection limits of them are not sufficient for direct detection of CT from stool sample. It was reported that CT concentrations in most of the patient stools were lower than 10 ng/mL, even though the concentrations of CT in patient stools vary, ranging from 26 pg/mL to >100 ng/mL [15]. For some toxigenic bacteria, including thermostable direct hemolysin- (TDH-) producing *V. parahaemolyticus *and Shiga toxin (Stx-) producing *E. coli*, it had been reported that precultivation by using enrichment media is helpful for immunochromatographic identification of toxigenic bacteria from stool or food sample [16, 17]. In the case of CT detection, alkaline peptone water (APW) is expected to be useful as the enrichment media. APW is most commonly used media for enrichment step in *Vibrio* spp. isolation [18]. In APW, *Vibrio* spp. can grow rapidly whereas growth of *E. coli* is inhibited or slow. This selectivity of APW has an important implication for CT detection because some ETEC have the ability to develop false-positive signal on CT-IC (Figure 3). So, if APW is employed at enrichment precultivation step, the occurrence of false-positive results might be reduced. In addition, it is evidence to the value of APW that *V. parahaemolyticus* did not give false-positive result in CT-IC. That is, even if *V. parahaemolyticus*, which is the most frequently isolated species among genus *Vibrio*, grow during enrichment cultivation in APW, it might have no effect on the results of CT-IC detection.

Various methods including immunoassay, bioassay, and DNA-based assay for investigation of toxigenicity of *V. cholerae* isolates had been established. Within them, immunoassay and bioassay require some degree of CT expression. Optimal *in vitro* CT expression in the El Tor biotype of *V. cholerae* serogroup O1 which is causative bacterium of ongoing 7th cholera pandemic had been persistently discussed because optimal condition for CT expression in El Tor biotype was significantly different from that for classical biotype which was responsible for the earlier cholera pandemics. Cultivation procedures with AKI media had been developed as a powerful tool for investigation of toxigenicity of *V. cholerae* isolates [3, 11–14]. It had been reported that most of *V. cholerae* El Tor could express considerably high amount of CT in the AKI media. In addition to AKI media, yeast extract-peptone water (YEP) media were reported to be able to stimulate CT expression, and the expression level of CT in YEP media tended to be higher than in AKI media [11]. YEP media differ from AKI media in the lack of sodium bicarbonate. Bicarbonate is an important component of small-intestinal fluid to protect intestine from acid arriving from stomach and is present in upper small intestine where *V. cholerae* colonizes at the almost the same concentration as in AKI media. Although some differences in molecular mechanism regulating CT expression between *in vitro* and *in vivo* had been reported, it was supposed that the expression level of CT in AKI media more closely mimic the situation in small intestine than in other media [19–21]. For this reason, we used AKI media in the present study. In addition, we employed “complex” biphasic AKI-SW condition to induce CT expression because CT expression under AKI-SW condition is considerably higher than that under monophasic culture condition (i.e., 20 h cultivation in a stationary test tube at 37°C). On the other hand, previous reports indicated that almost all of the *ct* gene-positive *V. cholerae* strains could express CT at concentration higher than the detection limit of CT-IC (10 ng/mL) even under the monophasic culture condition [11, 13]. In addition, we have confirmed that CT-IC could detect CT from 14 out of 15 toxigenic *V. cholerae* strains even under monophasic culture condition (data not shown). Based on these results, there is a strong possibility that CT-IC can detect almost all of toxigenic strain even under the monophasic culture condition. However, we think that cultivation with AKI-SW condition is indispensable for accurate judgment of toxigenicity because, for example, in our case, 1 out of 15 toxigenic strains was missed with monophasic culture condition. So, we suggest that, especially when a lot of samples must be examined, the “simple” monophasic stationary culture condition may be used for preliminary screening to reduce the number of samples. And only for the isolates gave negative results in the primary screening, further elaborate investigation must be done with “complex” AKI-SW condition to judge toxigenicity as accurately as possible.

Taken together, combined use of APW and AKI medium is predictably beneficial at the sample preparation for CT-IC. APW may help reduce false-positive result and AKI may help give accurate judgment of toxigenicity. Further studies are needed to investigate the ability of CT-IC to identify toxigenic *V. cholerae* from patient stools. 

## 5. Conclusions

In the case of *V. cholerae* identification, typing based on toxigenicity is critical. In this study, we established a toxigenic *V. cholerae*-specific immunochromatographic test strip. Because polyclonal antibodies were employed, the established test strip might have an ability to detect CT in various isolates even if some minor mutation in antigen was occurred in it. In addition to the adaptive capacity for polymorphism, the established test strip has high specificity; that is, it can discriminate CT from LT. We conclude that this high specific immunochromatographic strip is valuable in preventing the risk of failing to detect toxigenic *V. cholerae*.

## Figures and Tables

**Table 1:** Profiles of *V. cholerae* strains used in this study.

Year of isolation	Isolated from*	Serotype	*ct* gene
1993	Traveler's diarrhea case (India)	O1 El Tol Ogawa	+
1993	Traveler's diarrhea case (Philippines)	O1 El Tol Ogawa	+
1995	Traveler's diarrhea case (China)	O1 El Tol Ogawa	+
1996	Traveler's diarrhea case (Thailand)	O1 El Tol Ogawa	+
1996	Traveler's diarrhea case (Thailand)	O1 El Tol Ogawa	+
1996	Traveler's diarrhea case (Thailand)	O1 El Tol Ogawa	+
2006	Cholera patient lacking detailed information	O1 El Tol Ogawa	+
1997	Domestic case	O1 El Tol Inaba	+
2000	Traveler's diarrhea case (Thailand)	O1 El Tol Inaba	+
2001	Traveler's diarrhea case (China)	O1 El Tol Inaba	+
2004	Traveler's diarrhea case (Thailand)	O1 El Tol Inaba	+
2007	Traveler's diarrhea case (India)	O1 El Tol Inaba	+
2007	Traveler's diarrhea case (India)	O1 El Tol Inaba	+
1993	Traveler's diarrhea case (India)	O139	+
2004	Domestic case	O141	+
1998	Traveler's diarrhea case (Thailand)	O1 El Tol Ogawa	−
1999	Traveler's diarrhea case (Egypt, Greece)	O1 El Tol Ogawa	−
1997	Traveler's diarrhea case (Iran)	O1 El Tol Inaba	−
2001	Domestic case	O1 El Tol Inaba	−
2003	Cholera patient lacking detailed information	O139	−

*The places where the traveler's diarrhea patients were staying are indicated in brackets.
